# Sirtuin 3 deficiency does not impede digit regeneration in mice

**DOI:** 10.1038/s41598-019-52921-z

**Published:** 2019-11-11

**Authors:** Emily Busse, Jennifer Simkin, Luis Marrero, Kennon Stewart, Regina Brunauer, Ken Muneoka, Anyonya Guntur, Michelle Lacey, Mimi Sammarco

**Affiliations:** 1 Tulane School of Medicine, Department of Surgery, New Orleans, 70112 United States; 2 Louisiana State University Health Sciences Center, Department of Orthopedics, New Orleans, LA United States; 3 Tulane University, Department of Mathematics, New Orleans, LA United States; 4 Texas A&M University, Veterinary Physiology and Pharmacology, College Station, TX United States; 5 Maine Medical Center Research Institute, Center for Molecular Medicine, Scarborough, ME United States

**Keywords:** Targeted bone remodelling, Targeted bone remodelling, Molecular medicine, Molecular medicine

## Abstract

The mitochondrial deacetylase sirtuin 3 (SIRT3) is thought to be one of the main contributors to metabolic flexibility–promoting mitochondrial energy production and maintaining homeostasis. In bone, metabolic profiles are tightly regulated and the loss of SIRT3 has deleterious effects on bone volume *in vivo* and on osteoblast differentiation *in vitro*. Despite the prominent role of this protein in bone stem cell proliferation, metabolic activity, and differentiation, the importance of SIRT3 for regeneration after bone injury has never been reported. We show here, using the mouse digit amputation model, that SIRT3 deficiency has no impact on the regenerative capacity and architecture of bone and soft tissue. Regeneration occurs in SIRT3 deficient mice in spite of the reduced oxidative metabolic profile of the periosteal cells. These data suggest that bone regeneration, in contrast to homeostatic bone turnover, is not reliant upon active SIRT3, and our results highlight the need to examine known roles of SIRT3 in the context of injury.

## Introduction

The management of fuel resources and energy consumption by cells during times of anabolic and catabolic activity is critical to successful cell proliferation, differentiation, and function. In bone, bioenergetic programs are strictly regulated during both homeostasis and development^1^. For instance, pre-osteoblastic mesenchymal stem cells rely predominately on glycolytic metabolism during proliferation^2^, switch to increased cellular respiration and oxidative phosphorylation during differentiation and revert back to glycolytic metabolism after differentiation into osteoblasts^3^. The bioenergetics of osteoclasts have not been extensively studied and what results are reported are somewhat conflicting^4^. Recent studies suggest that osteoclasts use oxidative phosphorylation as the main energy pathway during differentiation and glycolytic pathways during bone resorption^5^. However, earlier studies suggest that osteoclasts utilize fatty oxidation during periods of bone resorption^6^. While it is clear that a more thorough investigation is necessary to fully understand the bioenergetics of osteoblasts and osteoclasts, these findings suggest that metabolic flexibility across several different bone cell lineages is required for successful bone turnover and specifically suggests metabolic flexibility would be essential to successful healing after bone injury.

Some of the main contributors to metabolic flexibility are the Sirtuins, a family of mitochondrial deacetylases. Sirtuin 3 (SIRT3), specifically, is the main mitochondrial deacetylase^7,8^ and promotes mitochondrial energy production and metabolic homeostasis^9^. SIRT3 mechanistically mediates metabolic switching in the cell by destabilizing hypoxia-inducible factor -1α (HIF1α), which transcriptionally promotes glycolytic gene expression^10^. The loss of SIRT3 results in increased reactive oxygen species (ROS), which in turn induce genomic instability and increase levels of HIF1α, promoting the preferential use of glycolytic pathways, otherwise known as the Warburg effect^11^. As a result, SIRT3 has been evaluated predominantly as a tumor suppressor. However, the integral role of SIRT3 in mediating metabolic switching has broadened the research interest in SIRT3. The loss of SIRT3 in knockout mice results in decreased bone volume likely due to increased numbers of osteoclasts^12,13^ that are directly linked to increased ROS^14^. This leads to decreased bone volume and trabecular number, and lower mechanical competence^13^. SIRT3 KO mice also demonstrate deficiencies in osteoblast differentiation^12,13,15^, and mature osteoblasts display reduced alkaline phosphatase activity, and decreased levels of expression of osteocalcin and RUNX2^13^.

Within the context of bone bioenergetics and the known interaction of SIRT3 on osteoblasts and osteoclasts, it is important to establish the role that SIRT3 plays *in vivo* in bone and soft tissue regeneration. We use a mammalian mouse model of tissue regeneration to test the hypothesis that SIRT3 is necessary for proper tissue repair after injury. In this mouse model of digit regeneration, the distal tip of the third phalangeal element (P3) is amputated. Following amputation, the mouse is able to mount a regenerative response. Soft tissue, epidermis, nail, and bone regrow in a distinct triangular pattern to replace all components of the damaged tissue^16–24^. After amputation there is an osteoclast-driven degradation event that excises the amputation plane, followed by epithelial wound closure, blastema formation, and patterned bone regrowth^16–19,25^. These phases are characterized by distinct populations of cells^26^ and the regenerative sequence is dependent upon a dynamic oxygen event^27,28^. This model provides us with three distinct advantages to test the role of SIRT3 after injury: (1) This model enables us to quantify complex tissue regeneration after injury using metrics to measure bone volume, pattern, and length; (2) Unlike long bone fracture healing models, regeneration after a P3 amputation occurs through intramembranous ossification, a process distinct from endochondral ossification of P3 development and (3) This model has a well characterized series of both degradative and regenerative events.

In this study, we confirm a role for SIRT3 in the metabolic function of periosteal osteoblasts by testing the metabolic capacity of osteoblasts isolated from SIRT3^−/−^ mice. We show that despite a reduction in metabolic capacity, SIRT3 knockout mice are able to regenerate bone and associated tissues after amputation injury. While SIRT3 has been shown to play an integral role in bone homeostasis^12,13,15^, our model is the first *in vivo* model to test the role of SIRT3 after bone injury. SIRT3 deficiency does not impede digit regeneration in mice and both the histolytic bone degradation phase of regeneration and the anabolic bone event progress with no major impact to timing, magnitude, or quality of the bone regenerate. Our findings in an *in vivo* bone and soft tissue regeneration model are critical to fully understanding the role of SIRT3 in bone anabolism and catabolism and highlight the need to examine known roles of sirtuins in the context of injury models.

## Results

### SIRT3 deficiency reduces oxidative and glycolytic capacity in periosteal osteoblasts

**Figure 1:**
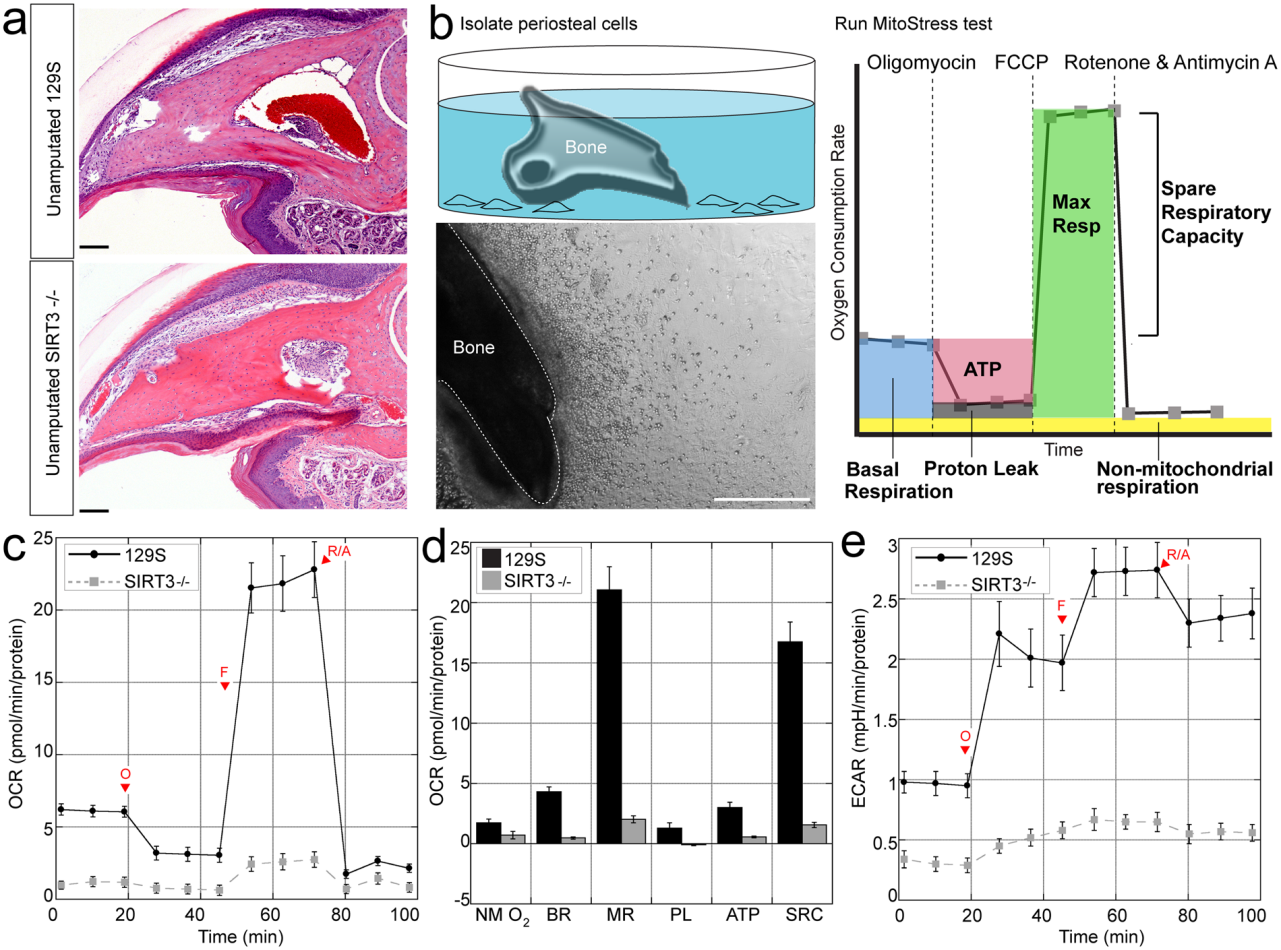
Periosteal cells from SIRT3^−/−^ mice show reduced metabolic capacity. (**a**) Hematoxlylin and eosin staining of the unamputated third phalangeal element (P3) bones of SIRT3^−/−^ and wildtype 129S littermates show no histological differences in the adult 8-week old mice. Scale bar = 50 μm. N = 4 with representative image shown. (**b**) We isolate P3 bones from knockout and wildtype littermates and allow periosteal cells to crawl onto a dish. Scale bar = 500 μm. (**c**) The P3 periosteal cells are assayed using the Seahorse Mito Stress Test. Changes in oxygen consumption rate are measured after injections of oligomyocin, FCCP, and antimycin A and rotenone. SIRT3^−/−^ cells show reduced oxygen consumption rate over time (OCR) compared to 129S littermates and (**d**) reduced non-mitochondrial oxygen consumption (NM O_2_), basal respiration (BR), maximal respiration (MR), proton leak (PL) ATP production (ATP), and spare respiratory capacity (SRC). All data were normalized to total protein. Error bars represent SEM (N = 10 wells). All comparisons are significant p < 0.05. (**e**) SIRT3^−/−^ cells show reduced extracellular acidification rate (ECAR) over time compared to 129S control cells.

Regeneration of the P3 bone is dependent upon periosteum-derived cells^29^, that show P3-specific behaviors including high proliferation and the ability to differentiate into osteoblasts^30^. Because the process of homeostatic bone turnover is dependent upon tightly controlled metabolic profiles of osteoblasts^12,13,15^, and because SIRT3 is responsible for metabolic flexibility^31^, we tested the metabolic profile of periosteum-derived cells from SIRT3 deficient mice and compared the metabolic profile to periosteum-derived cells from control mice expressing SIRT3. We isolated P3 bones from 8-week old mice (Fig. 1a) and removed the surrounding connective tissue, allowing the P3 periosteal cells to migrate off the explant over the course of two weeks^32^ (Fig. 1b). Cells from P3 bones obtained from SIRT3^−/−^ and 129S control mice were assayed using the Seahorse Mito Stress Test kit on the XFe24 Seahorse Analyzer (Fig. 1c). Using this assay we measured the basal respiration (the endogenous rate of respiration used to drive ATP synthesis and that associated with proton leak pathways), non-mitochondrial respiration (respiration presumably due to the activity of flavin-linked NAD(P)H oxidases^33^), maximal respiration (an indication of the spare respiratory capacity that the cell population holds in reserve), respiration associated with proton leak (protons that leak back across the mitochondrial inner membrane decreasing the membrane potential and stimulating upkeep of the membrane potential^34^), ATP-linked respiration (a rate that is indicative of ATP-utilization, ATP synthesis, and ATP substrate supply^35^), spare reserve capacity (the ability of a cell to respond to ATP demand and periods of stress^35^), and coupling efficiency (the proportion of respiratory activity that is used to make ATP, an indication of the coupling of oxidation to phosphorylation^36^). These parameters are identified using the ATP synthase inhibitor oligomycin, the potent mitochondrial uncoupler FCCP, and rotenone/antimycin A which block complex I and III^35^ (Fig. 1). Non-mitochondrial oxygen consumption (NMO_2_), basal respiration (BR), maximal respiration (MR), proton leak (PL), ATP production (ATP), and spare respiratory capacity (SRC) were all significantly reduced in P3 cells from the SIRT3^−/−^ cells as compared to 129S control cells (Fig. 1d). There was no significant difference in coupling efficiency between the two groups. Both the oxygen consumption rate (OCR) (Fig. 1c) and Extracellular Acidification Rate (ECAR) (Fig. 1e), a measure of glycolysis, were significantly reduced in SIRT3^−/−^ P3 cells.

### SIRT3^−/−^ has no impact on digit development

**Figure 2:**
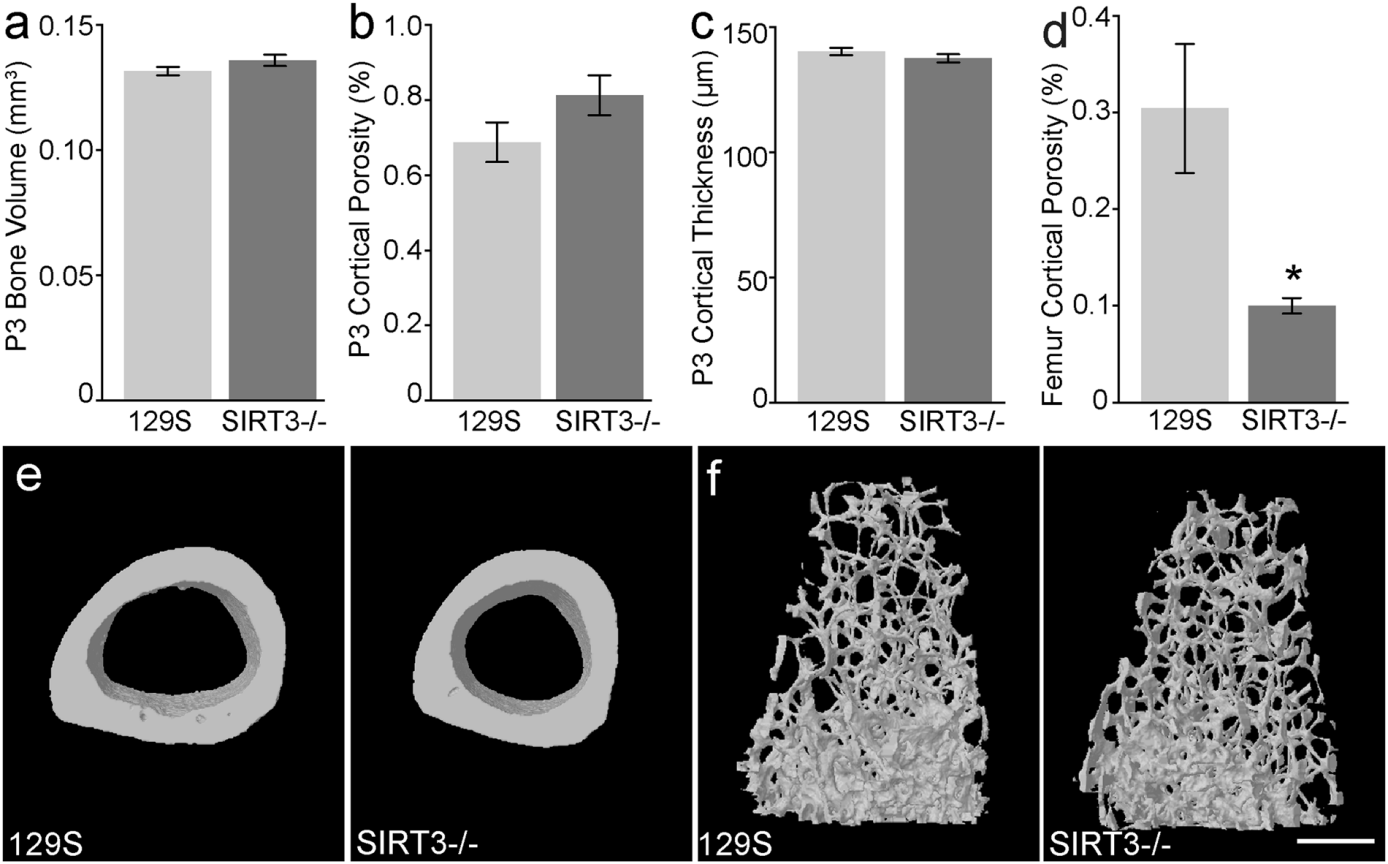
Femurs but not P3 bones of SIRT3^−/−^ mice show homeostatic defects. μCT analysis of the uninjured P3 bone reveals (**a**) total bone volume, (**b**) cortical porosity, and (**c**) cortical thickness are comparable between SIRT3^−/−^ and 129S wildtype littermates (N = 5 mice, N = 20 digits). (**d,e**) SIRT3^−/−^ femurs show decreased cortical porosity compared to 129S control mice. (**f**) Representative μCT images of the trabecular bone of the femur of adult 129S and SIRT3^−/−^ mice. N = 4 with representative sample shown. Scale bar represents 500 μm. Error bars represent SEM *p = 0.023.

The results that P3 cells from a SIRT3 deficient mouse showed reduced OCR and ECAR prompted us to analyze the *in vivo* regenerative capacity of these mice. Because SIRT3 has been shown to play a role in homeostatic bone turn-over, we first tested whether there were inherent differences in uninjured (unamputated) bone from SIRT3^−/−^ mice and 129S control mice. 8-week old SIRT3^−/−^ mice and 129S control mice were purchased directly from Jackson Labs and show the predicted truncated SIRT3 gene using standard published PCR primers from Jackson Laboratory (Supplemental Fig. 1). We analyzed P3 bones prior to amputation using a Scanco VivaCT 40 μCT. μCT analysis of unamputated digits show no significant differences between unamputated SIRT3^−/−^ and 129S P3 bones in bone volume (Fig. 2a), cortical porosity (Fig. 2b), cortical thickness (Fig. 2c). Histological analysis of unamputated SIRT3^−/−^ and 129S control digits show normal digit architecture (Fig. 1a). Prior publications^12,13,37^ indicated that SIRT3 deficiency produced an osteopenic phenotype and reduced bone mass in uninjured long bones. We therefore also analyzed the femur of SIRT3^−/−^ and 129S mice. μCT analysis of the trabecular bone of femurs in our study show that the SIRT3 KO samples have reduced cortical porosity (Fig. 2d,e); however, cortical bone volume, cortical thickness, and cortical tissue mineral density, as well as trabecular bone volume, trabecular thickness, trabecular separation, trabecular number, trabecular SMI, and trabecular bone mineral density showed no significant difference between SIRT3^−/−^ and 129S femurs. (Supplemental Fig. 2).

### SIRT3 deficiency has no major impact on the timing and rate of the regenerative phases of the digit

**Figure 3:**
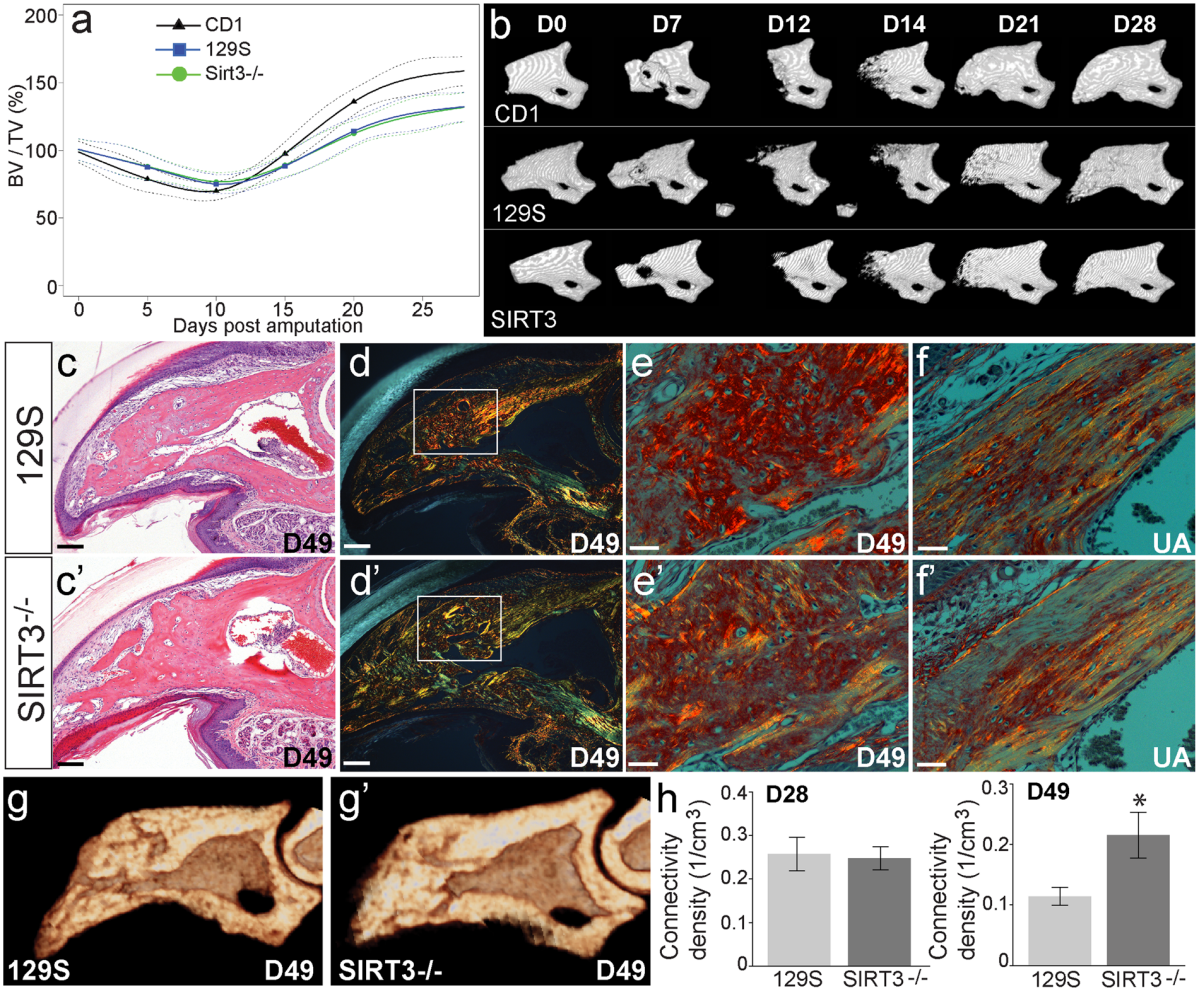
SIRT3 is not a limiting factor in digit regeneration. (**a**) μCT analysis of changes in bone volume over time following amputation shows no significant difference between SIRT3^−/−^ (green line circle) and 129S (blue line square) regenerative ability (N = 5 mice, N = 20 digits). CD1 outbred mice (black line triangle) are used as a standard amputation model for comparison (N = 4 mice, N = 16 digits). Data are normalized to amputated day 0 bone volume and analyzed using SS ANOVA models. (**b**) Representative μCT images of a regenerating P3 digit in CD1, SIRT3^−/−^, and 129S regenerates show no phenotypic differences at 28 days post amputation. (**c,c’**) H&E staining of day 49 sections of 129S (**c**) and SIRT3^−/−^ (**c’**) mice. Scale bar represents 50 μm. (**d,d’**) Picrosirius red staining and polarized light microscopy show collagen fiber alignment in woven bone of the regenerated digits at day 49 in 129S (**d**) and SIRT3^−/−^ (**d’**) mice. Scale bar represents 50 μm. (**e’**) Higher power images of d, d’ showing cross-hatched collagen alignment representative of woven bone in both 129S (**e**) and SIRT3^−/−^ (**e’**) regenerates. Scale bar represents 30 μm. (**f,f’**) For comparison, polarized light microscopy shows parallel collagen fiber alignment in the cortical bone of unamputated digits in 129S (**f**) and SIRT3^−/−^ (**f’**) mice. Scale bar represents 30 μm (**g,g’**) μCT cross-sectional analysis of regenerated digits at D49 to measure trabecular architecture in 129S and SIRT3^−/−^ mice. (**h**) Quantification of μCT data reveals trabeculae with greater connectivity density in the SIRT3^−/−^ mice compared to 129S controls at 49 days post amputation but not at 28 days post amputation. N = 4 for histology sections with representative images shown. Error bars represent SEM *p = 0.0162.

We next tested the ability of these mice to regenerate bone after amputation injury. Amputation of 129S control mice results in a regeneration response similar to that previously described for outbred CD1 mice and inbred C57BL6 mice^27,28^. Following this initial injury, an osteoclast driven bone degradation response occurs, resulting in a loss of 69.4%, 76.7%, and 74.9% of the amputated bone volume at day 10 for CD1, 129S control mice, and SIRT3 deficient mice respectively (Fig. 3a,b). Between 10 and 12 days after amputation, new bone starts to form and bone volume increases 158.7%, 131.8%, and 132% of the amputated volume by day 28 for CD1, 129S control mice, and SIRT3 deficient mice respectively (Fig. 3a,b). Similarly, amputation of the SIRT3^−/−^ digits results in a nearly identical response compared to controls (Fig. 3a,b). Both the volume and rate of bone degradation between day 0 and day 10 are identical to the control mice, and following the histolytic event, the rate of bone regeneration and final bone volume are comparable in mice deficient in SIRT3 and control mice (Fig. 3a). Histological analysis of regenerated digits from day 49 show complete wound closure, nail and soft tissue regeneration, and patterned bone regeneration (Fig. 3c,c’). In wildtype mice, regenerating bone is laid down as woven bone^38^. This woven bone can be distinguished from the cortical bone of the uninjured digit by how the collagen fibers are aligned. We sought to determine if loss of SIRT3 altered the type of bone (woven versus cortical) that was regenerated after amputation. One established technique for visualizing collagen fiber alignment in woven versus in cortical bone is picrosirius red staining and polarized light microscopy^39,40^. Woven bone takes on a cross-hatched fiber alignment, whereas cortical bone shows dense parallel fibers. Therefore, we used picrosirius red staining and polarized light microscopy to visually determine if regenerated bone was cortical or woven in the control and KO groups (Fig. 3d,e) Picrosirius red staining of samples from both the control and KO mouse groups show woven bone in the regenerates of both groups (Fig. 3d,e) compared to cortical bone of injured digits (3f,f’). Detailed μCT analysis of the fully regenerated bones 28 and 49 days after amputation show no significant difference from 129S controls in bone volume, trabecular thickness, trabecular separation, SMI, and trabecular number (Supplemental Fig. 3). However, SIRT3^−/−^ regenerated digits remodel the trabecular bone to show a significantly increased trabecular connectivity density when compared to 129S regenerated digits at day 49 (Fig. 3g,g’,h). Overall, these results suggest that SIRT3^−/−^ mice are able to regenerate digits comparable to the 129S controls.

### The influence of mouse strain and level of amputation on digit regeneration

**Figure 4:**
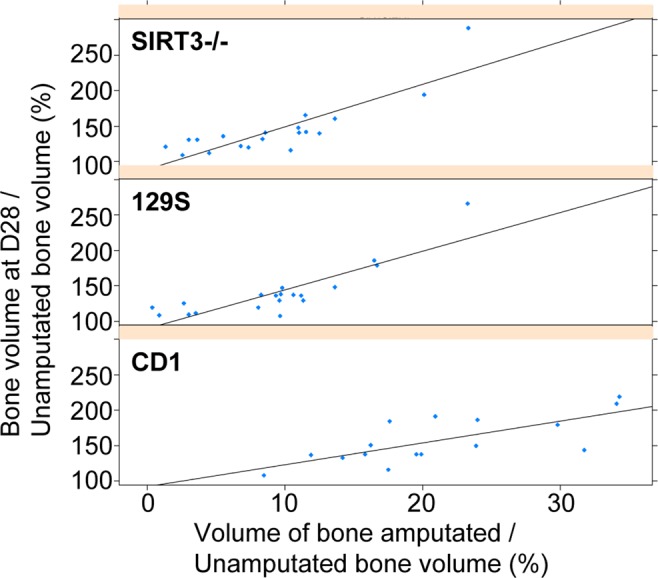
Level of amputation influences digit regeneration across mouse strains. Scatter plot showing the correlation between the volume of bone amputated and the amount of bone regeneration after amputation for SIRT3^−/−^, 129S, and CD1 mice with fitted regression lines. Amputation of approximately 10% of the bone volume results in 100% bone regrowth volume. Amputation of 20% or more of the original bone volume results in a final volume that exceeds the original bone volume. (N = 5 mice, N = 20 digits).

Interestingly, when the 129S control mice and SIRT3^−/−^ mice were compared to μCT bone volume data from CD1 outbred mice using SS ANOVA analysis, both strains of mice showed less bone degradation than the CD1 group (Fig. 3a). CD1 regeneration has been previously published^28^ and is used here as a reference for the regeneration profile of a wild type outbred mouse. While all three groups show similar timing transitioning from a catabolic to an anabolic bone event the CD1 group showed a faster rate of bone regeneration and a significantly larger final bone volume at day 28 (Fig. 3a). These differences indicate overall strain differences in the digit amputation model, underscoring the importance of the 129S control in this study. A closer analysis of bone amputation and regrowth in both the CD1, SIRT3^−/−^ mice, and 129S strains show that all three mouse strains demonstrate a statistically significant association between the level of amputation and the volume of regenerated bone (Fig. 4). Amputation planes that represent 10% or less of the unamputated bone show a regeneration volume that is approximately 100% of the original unamputated volume with a small amount of degradation and a small amount of regeneration in response. However, amputations that are representative of 20% or more of the original unamputated bone volume result in a regrowth volume that exceeds the original unamputated volume of bone (Fig. 4). Variability of amputation level was higher in the SIRT3^−/−^ mice and 129S mice than in the CD1 group, which allowed for closer scrutiny of the relationship between amputation level and bone regrowth volume.

## Discussion

Our data support that SIRT3 deficiency does not impede the process of bone and soft tissue regeneration in the mouse digit amputation model. Our findings are particularly relevant in the context of recent publications showing that SIRT3 is essential in bone homeostasis^12,14^, osteoblast differentiation^13^, and greatly influential in osteoclastogenesis^14,41^. Our findings are also pertinent in the context of recent focus on SIRT3 as a potential therapeutic target for certain cancers^42,43^, and support that this line of treatments would not compromise bone regeneration.

Given that the progression of an osteoblast from pre-osteoblast to osteocyte is dependent on a dynamic metabolic profile that incorporates both glycolytic pathways and oxidative pathways^1^, and data supporting SIRT3 deficiency results in a HIF1-α driven glycolytic profile^10,11,31,44,45^ we expected that SIRT3 knockout would attenuate the anabolic, and thus OxPhos-driven, bone regeneration phase both in timing and in bone volume. Furthermore, given that the SIRT3 knockout is universal, we also expected that the knockout would exacerbate the histolytic event by increasing bone resorption by the osteoclasts, which relies predominantly on glycolytic pathways^5^. However, both the catabolic and anabolic bone rates, volumes, and timing during regeneration were unaffected by the knockout of SIRT3. Additionally, histological evaluation of the regenerated digits showed that SIRT3 deficiency did not prevent wound closure nor result in overt phenotypic anomalies in the bone and soft tissue during regeneration.

SIRT3 deficiency had no detectable effect on the composition of the regenerated bone. While both samples showed an expected decrease in connectivity density from day 28 to 49 as a result of bone remodeling, which reduces the number of trabecular spaces, SIRT3 deficiency did unexpectedly increase connectivity density in the architecture of the regenerated digit as compared to the control. Increased connectivity density is structurally related to increased cortical bone strength^46^, where a well-connected network of trabecular bone protects cortical bone by diffusing damage through the trabecular network. Bone connectivity is considered to be a large component of bone strength^47–50^, and a practical indicator of bone quality^51^. With all other parameters held constant in comparison with the 129S control, this suggests that SIRT3 deficiency regenerated bone with better structural integrity than the control digit, however without additional evaluation this parameter alone is not able to speak conclusively to strength of bone, particularly in our model where this has not previously been evaluated. Our femur data showing reduced cortical porosity also supports better structural integrity. These findings agree with findings with other labs^12,13,37^ in the sense that SIRT3 does indeed impact bone architecture during development of the femur. All long bones, including the digit tip and femur, are formed via endochondral ossification, where the bone develops via a cartilage intermediate^52^. However, digit tip regeneration occurs via intramembranous ossification, with no cartilage intermediate. The trabecular bone analysis from the femur shows no differences from the control, which suggests that SIRT3 deficiency has a minimal effect on trabecular bone during development and that influence is restricted to regeneration of the woven bone.

While we did see decreased cortical porosity in the SIRT3^−/−^ femurs, other previously reported phenotypes, such as decreased trabecular thickness and bone mass^13^ were not evident. This may be attributed to differences in strains, as prior publications used independently generated SIRT3^−/−^ mice^37^ that were subsequently backcrossed with C57BL/6 mice^12^. Similarly, while Gao *et al*. evaluated the Jackson Laboratory mouse strain (Jackson Labs #027975) and also saw decreased bone mass and osteopenia, those findings were also generated after the strain was backcrossed to C57BL/6^13^. Ho *et al*. analyzed femurs from 6 month old SIRT3^−/−^ mice and saw no bone phenotype, hypothesizing that the osteopenic phenotype was age-dependent^41^, however taken together with the data presented here we suggest that the osteopenic phenotype may be strain-related, rather than age-dependent. Bone density is known to be variable among inbred strains of mice and underscores the importance of closer scrutiny for single gene knockout strains^53^. Thus, additional work in other strains is needed to definitively determine whether an osteopenic bone phenotype is affiliated with only certain strains of SIRT3^−/−^ mice. Our study is also limited by the fact that only female mice were analyzed and does not rule out the possibility, while small, that digit regeneration in male mice is impacted by SIRT3 deficiency while regeneration in female mice is not.

The lack of regenerative phenotype in the SIRT3^−/−^ mice is even more curious in light of our metabolic findings from the SIRT3^−/−^ cells showing both a reduced oxidative and glycolytic capacity. The 129S control P3 cells show a robust mitochondrial response suggesting strong ATP generation through oxidative phosphorylation. The increase in the FCCP-induced OCR suggests a potentially underutilized metabolic capacity in the uninjured P3 and our data show that control cells utilize both OCR and ECAR to meet ATP demand. Curiously SIRT3^−/−^ cells show almost no capacity to generate ATP, and the minimal response of ECAR to oligomycin may mean that glycolysis is at or approaching maximal capacity or limited by a low demand for ATP^35^. One explanation for our findings may be that cells are obtained from a model not under stress – that is the cells are isolated from an unamputated P3 bone as an assessment of the endogenous resting metabolic profile of the digit. It may be that SIRT3 deficient cells from an amputated bone under stress would show a different metabolic profile more similar to a control cell line under the same stress, however further studies at additional timepoints would be needed to identify metabolic compensation after injury as an explanation for having no phenotypic difference.

Interestingly our analysis of the three strains of mice (129S, SIRT3^−/−^, CD1) revealed a consistent and direct relationship to the amount of bone amputated and the amount of regenerated bone. The finding that amputation of greater than 20% of the original unamputated volume results in a volume of regenerated bone that exceeds the original volume was consistent across all three strains of mice. This finding, while not directly related to SIRT3, is very relevant to the digit regeneration field.

Our findings join a growing number of publications across different fields that represent a closer *in vivo* analysis of the role of SIRT3 in complex processes, such as regeneration. In the case of SIRT3’s influence on bone, it may be that molecular mechanisms discerned *in vitro* have attenuated impact when translated to an *in vivo* system, possibly due to overlapping and redundant functions within the sirtuin family. Digit regeneration is known to be heavily influenced by other systemic processes such as the inflammatory response of macrophages, which has been shown to be integral to coordinate mouse digit tip regeneration^54^. *In vivo* studies in the SIRT3^−/−^ mouse show SIRT3 deficiency had very little impact on the proliferation and cytokine production of macrophages^55^. It may be that given the integral role of inflammation in regeneration the intact macrophage response in the knockout strain may be more indicative of the role of SIRT3 in regeneration. Our data support the development of potential therapies that may target SIRT3^56^ with the anticipation that they would have little impact on bone regeneration. Our findings are important to both the digit regeneration field and in the developing field of sirtuin research in that they highlight the complexity of resolving a single gene function in the context of complex processes such as bone and soft tissue regeneration *in vivo*.

## Materials and Methods

### Ethics statement

All experiments were performed in accordance with the standard operating procedures approved by the Institutional Animal Care and Use Committee of Tulane University Health Sciences Center.

### Amputations and animal handling

Adult 8-week old^18^ female 129S1 wild type (002448) and SIRT3^−/−^ (012755) mice were purchased from Jackson Laboratory (Bar Harbor, ME). Mice were anesthetized with 1–5% isoflurane gas with continuous inhalation. The second and fourth digits of both hind limbs were amputated at the P3 distal level as described previously^19,27^ and regenerating digits were collected at day 49 for analysis. The third digit was used as an unamputated control.

### Tissue collection and histology

Digits were fixed in zinc-buffered formalin (Z-fix, Anatech, Battle Creek, MI). Bone was decalcified for 48 hours in a formic-acid based decalcifier (Decal I, Surgipath, Richmond, IL). Once decalcified, all samples were processed for paraffin embedding and sectioned at 4 µm onto glass slides. Sections were stained with either Mayer’s Hematoxylin and Eosin Y, or Picro-Sirius red stain (Sigma-Aldrich, St. Louis, MO) and mounted using permanent mounting medium (Fisher Scientific, Waltham, MA). Brightfield micrographs were captured using an Olympus DP72 camera mounted on an Olympus BX60 microscope with rotating stage (Olympus America Inc, Center Valley, PA). Polarized light microscopy was carried out with filters to provide circularly polarized illumination.

### Cell culture and metabolic assays

Periosteal cells were harvested from the uninjured P3 bones of 129S control and SIRT3 deficient mice at 8 weeks-old. The mice were euthanized and the P3 bone was dissected at the joint away from P2 and surrounding connective tissue was removed. Bones were placed in a 24-well plate in high glucose DMEM (Life Technology, Carlsbad, California) supplemented with 10% FBS. Explants were cultured for two weeks and cells migrated onto the cell culture surface. Cells were expanded in the 24-well plate, trypsinized and seeded at 20,000 cells per well on Seahorse 24-well culture plates for metabolic evaluation without further expansion. Cells were evaluated after 24 hours using the Seahorse Bioscience Mito Stress Test (Agilent, Santa Clara, CA) using the standard protocol after optimization of both cell seeding density and FCCP concentration. Final well concetrations for stressors were 1.0 μM oligomycin, 0.75 μM FCCP, and 0.5 μM rotenone/antimycin A. Each well was normalized to total protein (N = 10 wells per group).

### Micro-computed tomography (μCT)

*In vivo* μCT images were acquired using a VivaCT 40 (Scanco Medical AG, Brüttisellen, Switzerland) at 1000 projections per 180 degrees with a voxel resolution of 10.5 μm^3^, and energy and intensity settings of 55 kV and 145 μA. Integration time for capturing the projections was set to 200 ms using continuous rotation. Images were segmented using the BoneJ2^57^ (Version 1.4.3) in conjunction with ImageJ 1.51 s. We utilized the Optimize Threshold Plugin for thresholding. 3D renderings of the μCT scans were created using the 3D viewer plugin for ImageJ. Images of the femurs were acquired using a SkyScan 1172 scanner (Bruker, Kontich, Belgium) at 55 kV and 181 µA, with 2 K resolution and a pixel size of 7.7 µm. Images were captured at a rotation angle of 0.4 with frame averaging of 2. The bottom boundary of the trabecular region of interest was 0.255 mm above the distal femoral growth plate. The top boundary of the trabecular region of interest was 2.45 mm above the bottom boundary. The cortical region of interest began 5 mm above the distal femoral growth plate for a total length of 1.15 mm. Raw images were processed with NRecon and Data Viewer. The trabecular and cortical regions of interest were each globally segmented for analysis and 3D modeling with CTAn. Tissue mineral density and bone mineral density were calculated using 2 mm density phantoms.

### Statistical analysis

Volume measurements for each digit were analyzed for the time period following amputation, with measurements expressed in terms of the volume percentage relative to the post-amputation baseline. Mixed effected Smoothing Spline Analysis of Variance (SS ANOVA) models^28,58,59^ were fit to the collected data to account for the nonlinearity of the growth trajectories and correlation in the repeated measurements for each animal and amputated digit. Analyses were performed using the R package ASSIST^60^ (Version 3.1.2), which incorporates algorithms to estimate the curves and approximate Bayesian 95% confidence intervals for the average growth levels for each treatment group. Analysis of *in vivo* μCT data using the smoothing spine ANOVA (SS ANOVA) algorithm described previously^28^ validates traditional T-test analyses, accounts for repeated sampling, and enables prediction of expected bone volume changes at all experimental time points. The smoothing spline ANOVA also provides continuous confidence intervals that establish useful limits of differences between groups.

Bone morphometrics from SIRT3^−/−^ and 129S control mice were assessed for differences via unpaired *t* tests with one-tailed or two-tailed distributions using KaleidaGraph (version 4.1.1; Synergy Software, Reading, PA. USA) and GraphPad (version 7.0d; Prism 7, San Diego, CA. USA). Unpaired tests were also conducted for histological analysis of unpaired digits. A value of *p* < 0.05 was deemed statistically significant. In all cases data are represented as mean ± SEM.

To analyze differences in degradation and regrowth profiles for amputated digits, volume measurements for each digit were analyzed for the time period following amputation, with measurements expressed in terms of the volume percentage relative to the post-amputation baseline. Mixed effects Smoothing Spline Analysis of Variance (SS ANOVA) models^28,58,59^ were fit to the collected data to account for the nonlinearity of the growth trajectories and correlation in the repeated measurements for each animal and amputated digit. Analyses were performed using the R package ASSIST^60^ (Version 3.4.3), which incorporates algorithms to estimate the curves and approximate Bayesian 95% confidence intervals for the average growth levels for each treatment group at each time point. Simple linear regression models were fit to assess the relationship between amputation depth and regrowth volume.

## Supplementary information

Sirtuin 3 deficiency does not impede digit regeneration in mice

## Data Availability

Data sets generated and/or analyzed during the current study are available from the corresponding author on reasonable request.

## References

[1] Riddle RC, Clemens TL (2017). Bone Cell Bioenergetics and Skeletal Energy Homeostasis. Physiol Rev.

[2] Shum LC, White NS, Mills BN, Bentley KL, Eliseev RA (2016). Energy Metabolism in Mesenchymal Stem Cells During Osteogenic Differentiation. Stem Cells Dev.

[3] Guntur AR, Le PT, Farber CR, Rosen CJ (2014). Bioenergetics during calvarial osteoblast differentiation reflect strain differences in bone mass. Endocrinology.

[4] Arnett TR, Orriss IR (2018). Metabolic properties of the osteoclast. Bone.

[5] Lemma S (2016). Energy metabolism in osteoclast formation and activity. Int J Biochem Cell Biol.

[6] Dodds RA, Gowen M, Bradbeer JN (1994). Microcytophotometric analysis of human osteoclast metabolism: lack of activity in certain oxidative pathways indicates inability to sustain biosynthesis during resorption. J Histochem Cytochem.

[7] Lombard DB (2007). Mammalian Sir2 homolog SIRT3 regulates global mitochondrial lysine acetylation. Mol Cell Biol.

[8] Yang W (2016). Mitochondrial Sirtuin Network Reveals Dynamic SIRT3-Dependent Deacetylation in Response to Membrane Depolarization. Cell.

[9] Finley LW, Haigis MC (2012). Metabolic regulation by SIRT3: implications for tumorigenesis. Trends Mol Med.

[10] Finley LW (2011). SIRT3 opposes reprogramming of cancer cell metabolism through HIF1alpha destabilization. Cancer Cell.

[11] Haigis MC, Deng CX, Finley LW, Kim HS, Gius D (2012). SIRT3 is a mitochondrial tumor suppressor: a scientific tale that connects aberrant cellular ROS, the Warburg effect, and carcinogenesis. Cancer Res.

[12] Huh JE (2016). Sirtuin 3 (SIRT3) maintains bone homeostasis by regulating AMPK-PGC-1beta axis in mice. Sci Rep.

[13] Gao J (2018). SIRT3/SOD2 maintains osteoblast differentiation and bone formation by regulating mitochondrial stress. Cell death and differentiation.

[14] Kim H, Lee YD, Kim HJ, Lee ZH, Kim HH (2017). SOD2 and Sirt3 Control Osteoclastogenesis by Regulating Mitochondrial ROS. Journal of bone and mineral research: the official journal of the American Society for Bone and Mineral Research.

[15] Ding Y (2017). Sirtuin 3 is required for osteogenic differentiation through maintenance of PGC-1a-SOD2-mediated regulation of mitochondrial function. Int J Biol Sci.

[16] Bryant SV, Endo T, Gardiner DM (2002). Vertebrate limb regeneration and the origin of limb stem cells. The International journal of developmental biology.

[17] Brockes JP, Kumar A (2005). Appendage regeneration in adult vertebrates and implications for regenerative medicine. Science.

[18] Han M, Yang X, Lee J, Allan CH, Muneoka K (2008). Development and regeneration of the neonatal digit tip in mice. Dev. Biol..

[19] Fernando WA (2011). Wound healing and blastema formation in regenerating digit tips of adult mice. Dev. Biol..

[20] Said S, Parke W, Neufeld DA (2004). Vascular supplies differ in regenerating and nonregenerating amputated rodent digits. The anatomical record. Part A, Discoveries in molecular, cellular, and evolutionary biology.

[21] Douglas BS (1972). Conservative management of guillotine amputation of the finger in children. Aust. Paediatr. J..

[22] Borgens RB (1982). Mice regrow the tips of their foretoes. Science.

[23] Illingworth CM (1974). Trapped fingers and amputated finger tips in children. J. Pediatr. Surg..

[24] Singer M, Weckesser EC, Geraudie J, Maier CE, Singer J (1987). Open finger tip healing and replacement after distal amputation in rhesus monkey with comparison to limb regeneration in lower vertebrates. Anatomy and embryology.

[25] Simkin J, Han M, Yu L, Yan M, Muneoka K (2013). The mouse digit tip: from wound healing to regeneration. Methods Mol Biol.

[26] Rinkevich Y, Lindau P, Ueno H, Longaker MT, Weissman IL (2011). Germ-layer and lineage-restricted stem/progenitors regenerate the mouse digit tip. Nature.

[27] Sammarco MC (2014). Endogenous bone regeneration is dependent upon a dynamic oxygen event. J. Bone Miner. Res..

[28] Sammarco MC (2015). Hyperbaric Oxygen Promotes Proximal Bone Regeneration and Organized Collagen Composition during Digit Regeneration. PloS one.

[29] Dawson LA (2017). The periosteal requirement and temporal dynamics of BMP2-induced middle phalanx regeneration in the adult mouse. Regeneration (Oxf).

[30] Wu Y (2013). Connective tissue fibroblast properties are position-dependent during mouse digit tip regeneration. PLoS One.

[31] Dittenhafer-Reed KE (2015). SIRT3 mediates multi-tissue coupling for metabolic fuel switching. Cell Metab.

[32] Duchamp de Lageneste O (2018). Periosteum contains skeletal stem cells with high bone regenerative potential controlled by Periostin. Nat Commun.

[33] Bedard K, Krause KH (2007). The NOX family of ROS-generating NADPH oxidases: physiology and pathophysiology. Physiol Rev.

[34] Jastroch M, Divakaruni AS, Mookerjee S, Treberg JR, Brand MD (2010). Mitochondrial proton and electron leaks. Essays Biochem.

[35] Divakaruni AS, Paradyse A, Ferrick DA, Murphy AN, Jastroch M (2014). Analysis and interpretation of microplate-based oxygen consumption and pH data. Methods Enzymol.

[36] Conley KE (2016). Mitochondria to motion: optimizing oxidative phosphorylation to improve exercise performance. J Exp Biol.

[37] Ahn BH (2008). A role for the mitochondrial deacetylase Sirt3 in regulating energy homeostasis. Proceedings of the National Academy of Sciences of the United States of America.

[38] Simkin J (2015). The mammalian blastema: regeneration at our fingertips. Regeneration (Oxf).

[39] Ip V, Toth Z, Chibnall J, McBride-Gagyi S (2016). Remnant woven bone and calcified cartilage in mouse bone: differences between ages/sex and effects on bone strength. PLoS One.

[40] Boerckel JD (2012). Effects of *in vivo* mechanical loading on large bone defect regeneration. J. Orthop. Res..

[41] Ho L (2017). Sirtuin-3 Promotes Adipogenesis, Osteoclastogenesis, and Bone Loss in Aging Male Mice. Endocrinology.

[42] Xiong Y (2017). SIRT3 is correlated with the malignancy of non-small cell lung cancer. Int J Oncol.

[43] Zeng H, Li L, Chen JX (2014). Loss of Sirt3 limits bone marrow cell-mediated angiogenesis and cardiac repair in post-myocardial infarction. PloS one.

[44] de Moura MB, Uppala R, Zhang Y, Van Houten B, Goetzman ES (2014). Overexpression of mitochondrial sirtuins alters glycolysis and mitochondrial function in HEK293 cells. PloS one.

[45] Schumacker PT (2011). SIRT3 controls cancer metabolic reprogramming by regulating ROS and HIF. Cancer Cell.

[46] Parfitt AM (1987). Trabecular bone architecture in the pathogenesis and prevention of fracture. Am J Med.

[47] Ulrich D, van Rietbergen B, Laib A, Ruegsegger P (1999). The ability of three-dimensional structural indices to reflect mechanical aspects of trabecular bone. Bone.

[48] Ciarelli TE, Fyhrie DP, Schaffler MB, Goldstein SA (2000). Variations in three-dimensional cancellous bone architecture of the proximal femur in female hip fractures and in controls. Journal of bone and mineral research: the official journal of the American Society for Bone and Mineral Research.

[49] Borah B (2002). Risedronate preserves trabecular architecture and increases bone strength in vertebra of ovariectomized minipigs as measured by three-dimensional microcomputed tomography. Journal of bone and mineral research: the official journal of the American Society for Bone and Mineral Research.

[50] Genant HK (2007). Severity of vertebral fracture reflects deterioration of bone microarchitecture. Osteoporos Int.

[51] Genant HK, Jiang Y (2006). Advanced imaging assessment of bone quality. Ann N Y Acad Sci.

[52] Berendsen AD, Olsen BR (2015). Bone development. Bone.

[53] Beamer WG, Donahue LR, Rosen CJ, Baylink DJ (1996). Genetic variability in adult bone density among inbred strains of mice. Bone.

[54] Simkin J (2017). Macrophages are required to coordinate mouse digit tip regeneration. Development.

[55] Ciarlo E (2017). Sirtuin 3 deficiency does not alter host defenses against bacterial and fungal infections. Sci Rep.

[56] Chiu HC (2018). Preventing muscle wasting by osteoporosis drug alendronate *in vitro* and in myopathy models via sirtuin-3 down-regulation. J Cachexia Sarcopenia Muscle.

[57] Doube M (2010). BoneJ: Free and extensible bone image analysis in Image. J. Bone.

[58] Wang Y (1998). Mixed-Effects Smoothing Spline ANOVA. Journal of the Royal Statistical Society B.

[59] Wang, Y. *Smoothing Splines: Methods and Applications*. 1 edn, (Chapman and Hall/CRC, 2011).

[60] ASSIST: A Suite of S-plus functions Implementing Spline smoothing Techniques v. R package Version 3.1.2. (2009).

