# Supplementary material for: Sirtuin 3 deficiency does not impede digit regeneration in mice
Source: Sci Rep. 2019 Nov 11;9:16491. doi: 10.1038/s41598-019-52921-z (PMC6848098; doi:10.1038/s41598-019-52921-z)
Supplement: Supplementary file 1 — Sirtuin 3 deficiency does not impede digit regeneration in mice [file 41598_2019_52921_MOESM1_ESM.pdf]

Sirtuin 3 deficiency does not impede digit regeneration in mice

Emily Busse[1], BS; Jennifer Simkin[2], PhD; Luis Marrero[2], PhD; Kennon Stewart[3]; Regina Brunauer[4], PhD; Ken Muneoka[4], PhD; Anyonya Guntur[5], PhD; Michelle Lacey[3], PhD; Mimi Sammarco[1*], PhD

Supplemental data

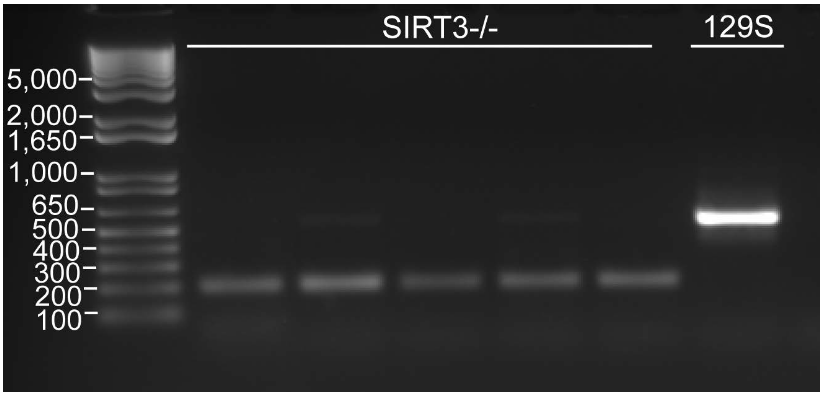

Supplemental Figure 1. DNA was isolated from the calf of SIRT3$^{-/-}$ and 129S control mice and amplified with PCR using primers published by Jackson Laboratory. The resulting fragments were separated using a 1.4% agarose gel and visualized using ethidium bromide. SIRT3$^{-/-}$ mice show a band at 200 bp indicating absence of SIRT3 gene. 129s control mice show a single band at 562 bp indicating presence of the SIRT3 gene. 1Kb Plus DNA ladder shown.

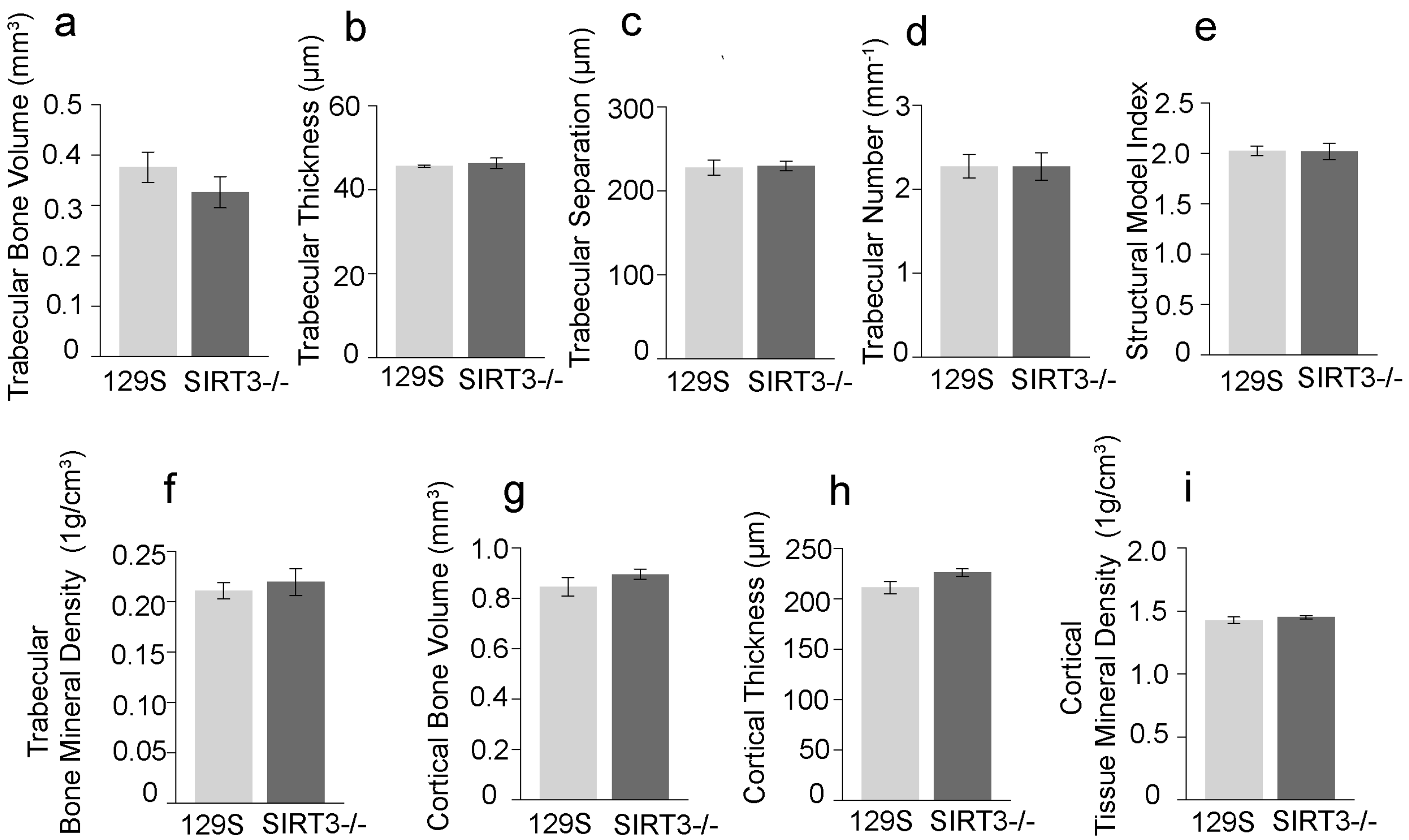

Supplemental Figure 2. Femur morphometrics for SIRT3$^{-/-}$ and 129S control mice based on µCT analysis – a) trabecular bone volume, b) trabecular thickness, c) trabecular separation, d) trabecular number, e) structural model index, f) trabecular bone mineral density, g) cortical bone volume, h) cortical thickness, and i) cortical tissue mineral density. Error bars represent SEM, N=4

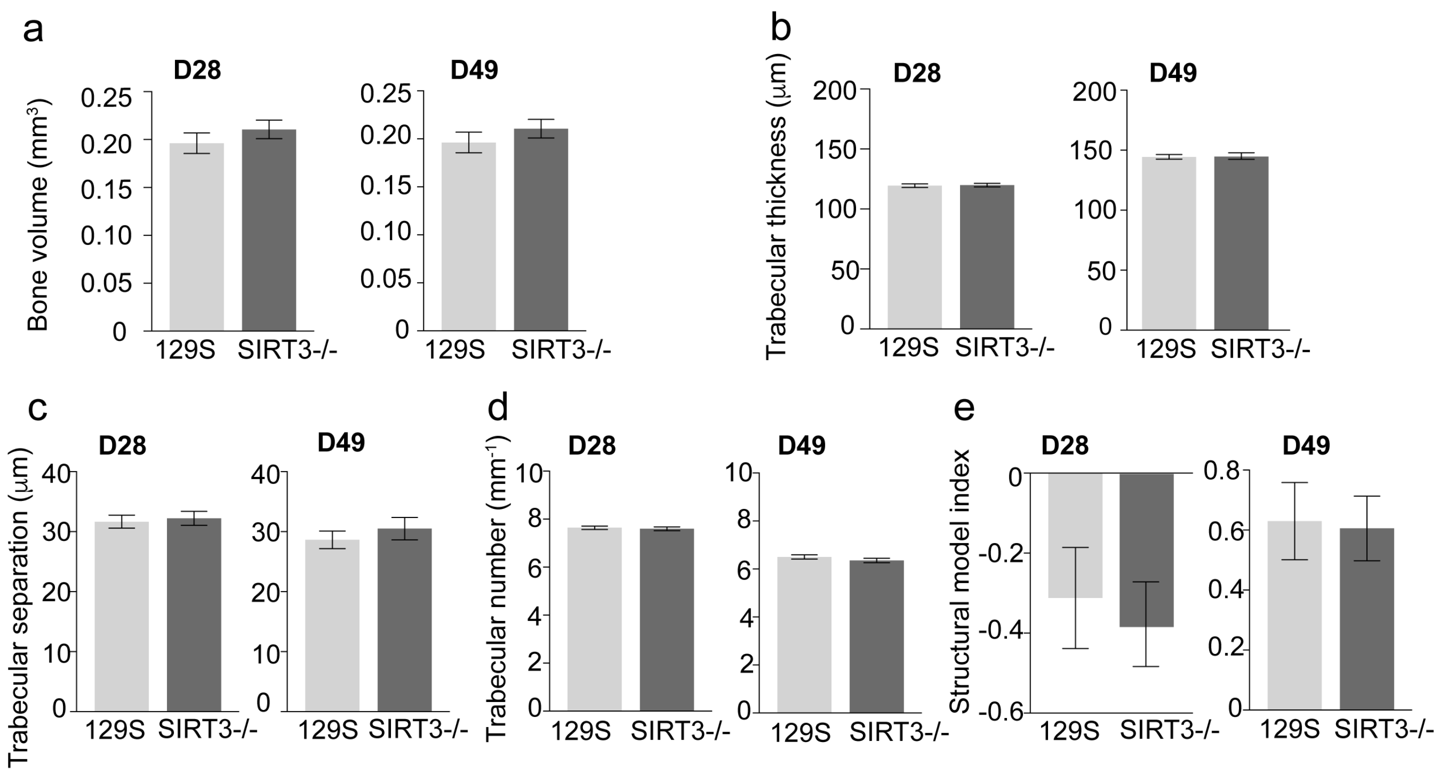

Supplemental Figure 3. Morphometrics for regenerated P3 bone at day 28 and 49 for SIRT3$^{-/-}$ and 129S control mice based on μCT analysis – a) bone volume, b) trabecular thickness, c) trabecular separation, d) trabecular number, and e) structural model index. Error bars represent SEM, N=5 mice, N=20 digits.
